# The utilisation of a structured debriefing framework within the pre-hospital environment: a service evaluation

**DOI:** 10.29045/14784726.2018.06.3.1.8

**Published:** 2018-06-01

**Authors:** Shaun Tierney

**Affiliations:** North West Ambulance Service NHS Trust

**Keywords:** clinical error, debrief, human factors, pre-hospital, team dynamics

## Abstract

**Background:**

Debriefing improves care and reduces error. To be effective, debriefs should be facilitated by trained individuals utilising structured and validated tools. Currently, in UK ambulance services there is no published evidence that structured processes utilising validated tools are being consistently delivered by trained facilitators, potentially impacting clinical practice.

**Methods:**

A pre-intervention survey related to debriefing was sent to 1000 clinicians within a specific geographical area of the trust via e-mail. In addition, 12 senior or advanced paramedics were recruited from the same area to participate in a training day and 12-week trial, utilising the Debrief Diamond as part of post-event debriefing. Following the trial period, all facilitators and participants of any recorded debriefs were invited to complete a post-intervention survey.

**Results:**

A total of 130 staff responded to the pre-intervention survey, with 22% reporting that previous debriefs had not identified areas for learning, and 13% reporting that previous debriefs had not identified good practice, learning opportunities or near misses. Post-intervention, 89% believed the process of debriefing was improved utilising a structured framework, 85% stated trained individuals improved the process, 93% reported the identification of good practice, 70% identified team level learning and 100% of facilitators reported improvements in identifying and supporting learning.

**Conclusion:**

Improvements in identifying good practice and learning opportunities were reported by both clinicians and facilitators in this evaluation, reflecting current evidence that structured and facilitated debriefs support safer care through the identification and subsequent reduction of human error. Consequently, the evaluation of appropriate debrief frameworks to provide consistency and validity to clinical debriefs in the pre-hospital environment should be considered to support safer clinical care.

## Introduction

Mistakes within healthcare result in human and economic costs. Individual patients and their families may suffer physical and/or mental harm and distress, or in extreme cases, life changing harm or even death may occur (Department of Health, 2015). These mistakes can also affect the healthcare professionals involved, leading to the ‘second victim’ phenomenon where feelings of shame, guilt or depression can be experienced, with the potential to end a clinician’s career (Seys et al., 2013).

In addition to the human cost of clinical error is the economic impact upon healthcare systems (NHS Litigation Authority, 2014). Analysis indicates preventable harm occurring in 5% of NHS care, with extrapolated costings of up to £3 billion (Frontier Economics, 2014). These estimates do not include the cost of litigation paid out by the NHS for preventable adverse events, with £1.4 billion being paid to patients and their legal representatives in 2014–2015 (NHS Litigation Authority, 2014).

A significant contributing factor in clinical errors is team dynamics, one of the human factors (also known as ergonomics). Shared mental models, objectives, communication and expectations are four aspects that are required for effective teamworking (The National Quality Board, 2013). In addition, briefing, debriefing and team preparation are advocated as best practice to optimise team performance (Carthey & Clarke, 2013; NHS Institute for Innovation and Improvement, 2010; Runnacles et al., 2014; Sandhu et al., 2014).

However, delivering these elements to support success within teams presents the ambulance service with genuine challenges. The structure of pre-hospital care in the UK results in the composition and membership of its teams often being unpredictable in numbers, skillset and experience (Shields & Flin, 2012). This unpredictability and lack of consistency in team structures, along with incidents being dynamic and unplanned, means briefing teams prior to incidents is usually not an option for ambulance services. However, there is an opportunity to generate effective learning through reflective processes following events, using structured debriefing (Kessler, Cheng, & Mullan, 2015).

The empirical and theoretical evidence examining debriefing is scant, spread thinly across numerous disciplines and lacking common reference points (Dufrene & Young, 2014; Dufresne, 2007; Tannenbaum & Cerasoli, 2013). In addition, most published research has been conducted during simulation, as opposed to post-event debriefing in a clinical setting (Cheng et al., 2015; Dufrene & Young, 2014; Sandhu et al., 2014). However, the results are encouraging, with debriefing being shown to demonstrate improvements to individual and team performance by up to 25% compared to those that do not utilise this form of learning (Mullan, Kessler, & Cheng, 2014; Sawyer, Loren, & Halamek, 2016; Tannenbaum & Cerasoli, 2013). Learning through debriefs is more effective and demonstrates improved outcomes when structured and facilitated (Ahmed et al., 2013; Jaye, Thomas, & Reedy, 2015; Runnacles et al., 2014; Tannenbaum & Cerasoli, 2013; Zigmont, Kappus, & Sudikoff, 2011). Conversely, poorly conducted debriefings can have negative consequences to learning and practice (Palaganas, Fey, & Simon, 2016).

Leading experts in the field of healthcare education have called for explicit models of debriefing to be developed, but despite this, only a few examples have been published (Jaye et al., 2015), and there is limited empirical evidence to support the use of any models following actual clinical events (Kessler et al., 2015). Debriefing is a complex and dynamic skill (Cheng et al., 2015), and using an evidence-based tool or script can help guide facilitators and teams through a structured process, particularly debrief novices (Kessler et al., 2015). Structured post-event debriefing should be distinguished from stress debriefing – often termed ‘defusing or critical incident debriefing’ – which is aimed at addressing emotions and psychological welfare, whereas clinical debriefing aims to improve future performance (Zigmont et al., 2011).

Debriefing crews following incidents such as cardiac arrest and major trauma is a core aspect of the role of senior paramedics (SPs) and advanced paramedics (APs) within the North West Ambulance Service (as seen in advanced paramedic and senior paramedic team leader job descriptions). However, results of an internal survey confirmed that within the North West Ambulance Service there is currently no defined, validated or structured process to learn from clinical events via the utilisation of a consistent and agreed debriefing process; this situation is mirrored nationally within NHS ambulance services in England (Tierney, 2017). This suggests the current position may be contributing to missed opportunities to highlight and reinforce good practice, with an associated failure to recognise areas for improving practice and identifying near misses.

## Aims

This evaluation aims to assess the introduction of the ‘Debrief Diamond’ (Figure 1 and 2), a validated debriefing framework (Jaye et al., 2015), and its impact upon learning from clinical events at individual, team and organisational levels. This framework was selected following a review of published and validated frameworks (Supplementary 1).

**Figure:**
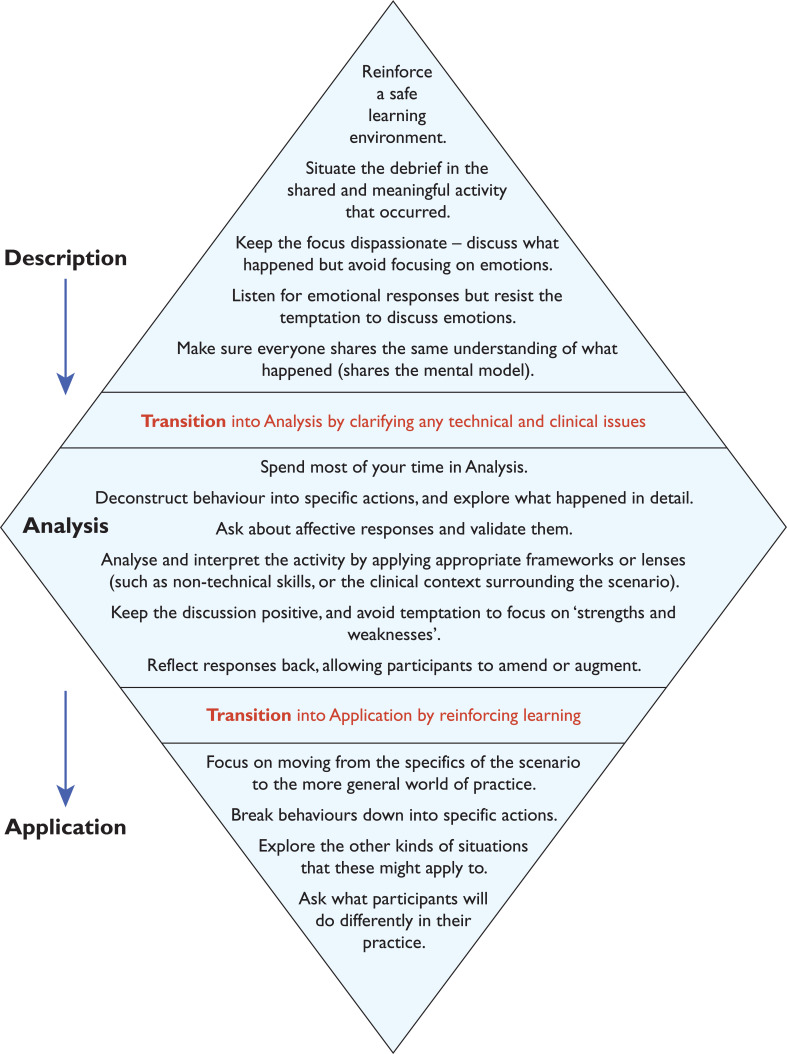
Figure 1. The Debrief Diamond: underlying principles.

Source: Jaye et al., 2015.

**Figure:**
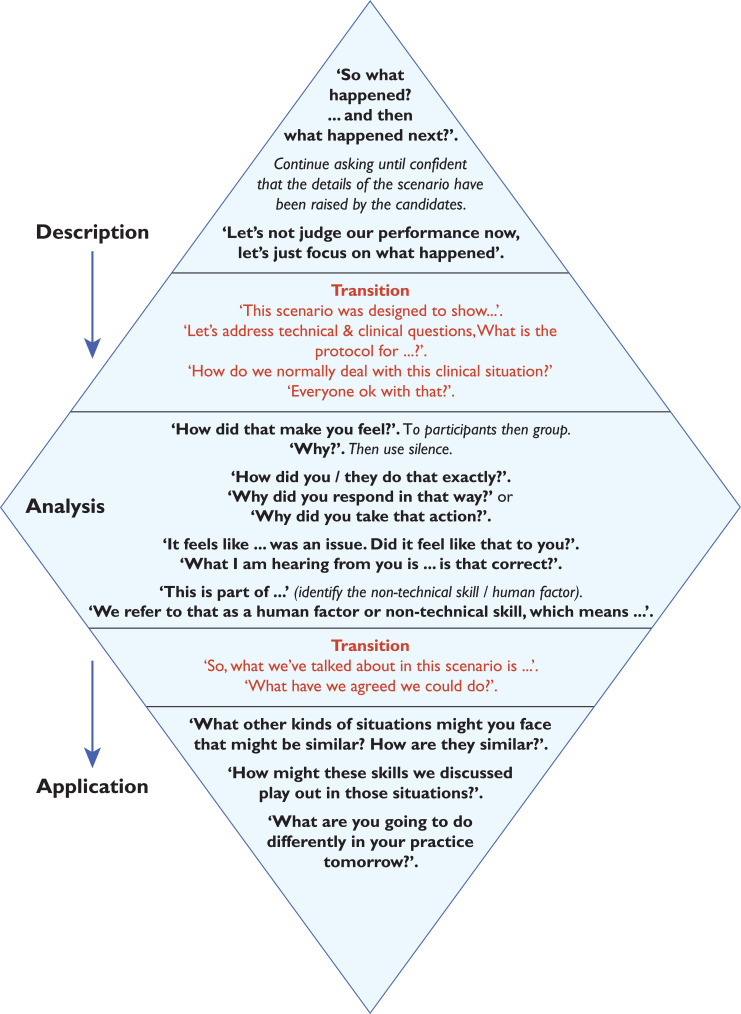
Figure 2. The Debrief Diamond: key phrases to remember.

Source: Jaye et al., 2015.

## Methods

The initial aspect of this study was to analyse staff experiences of debriefing within the organisation, followed by analysis post-intervention. The methodology chosen for both pre- and post-implementation was surveys, enabling the collection of information from a sample of the population of interest. Pre-intervention, a retrospective, cross-sectional survey (Aday & Cornelius, 2006) was carried out. Post-intervention, a ‘trend survey’ was applied, enabling identification of sample members who had been exposed to the intervention (Bowling, 2014).

### Pre-intervention

In December 2016, 1000 clinicians within a specific geographical area of the trust were e-mailed a link to the questionnaire and invited to participate, with a time limit of six weeks. Additional measures to support participation were a poster campaign, and championing of the study through local management teams. No incentives were offered for participation. The pre-intervention survey received a response rate of 130, equating to 13%; this resultant response rate of 13% offered an error margin of 8% (95% confidence level).

### Debrief facilitator recruitment

E-mails were distributed to all SPs and APs within the same geographical area via a third party, requesting volunteers to participate in the 12-week trial and an associated training day. The training comprised of theory and practical learning relating to human factors, debriefing and utilisation of the chosen tool. A total of 16 SPs and six APs (50% of the total SP and AP area population) were recruited; however, due to sickness and unforeseeable commitments only 12 SPs and five APs completed the training. Ensuring the recruited SPs and APs were familiar with the tool’s associated theory and use was achieved through its application in a simulated cardiac arrest scenario, with peer assessment utilising the Objective Structured Assessment of Debriefing (OSAD), a guide aimed at supporting and improving debriefs (Arora et al., 2012). During the trial period of January–March 2017, SPs and APs completed debriefs for any cardiac arrest or major trauma incident, as defined by the North West Ambulance Service Adult and Children’s Major Trauma Pathfinder tools (North West Ambulance Service, 2015a, 2015b), that they had been directly involved in or requested to attend for the purpose of performing a debrief. All staff participating were required to provide consent to participate and to be contacted post-intervention.

### Post-intervention

Following the 12-week trial, all facilitators and participants of any recorded debriefs were e-mailed a link to a post-implementation survey.

## Results

### Pre-intervention

Of the 130 respondents, 37% (48/130) did not complete the demographic section; of those that did, respondents were represented across a range of clinical and managerial grades, the largest group (40%, 33/83) having over 15 years’ front line experience. A total of 75% (97/130) had previously received a debrief across a range of incidents including cardiac arrests, major trauma, road traffic collisions, serious medical and capacity to consent incidents.

A total of 8% (8/99) of respondents to the question pertaining to debriefs being structured and facilitated by a trained individual did not believe this was a requirement, with comments such as ‘any emotionally intelligent individual being capable’ recorded, with SPs being among this group of respondents.

A total of 22% (22/100) did not feel that the identification of learning for improvement was identified during previous debriefs; 13% (13/100) felt neither good practice, opportunities for learning nor near misses were identified as part of previous debriefs. A number of respondents (7–14%, 7–14/101; Figure 3) disagreed that debriefs were important for identifying good practice or for learning at individual, team and organisational levels, with 18% (18/101) neither agreeing or disagreeing with these aspects in relation to debriefs.

**Figure:**
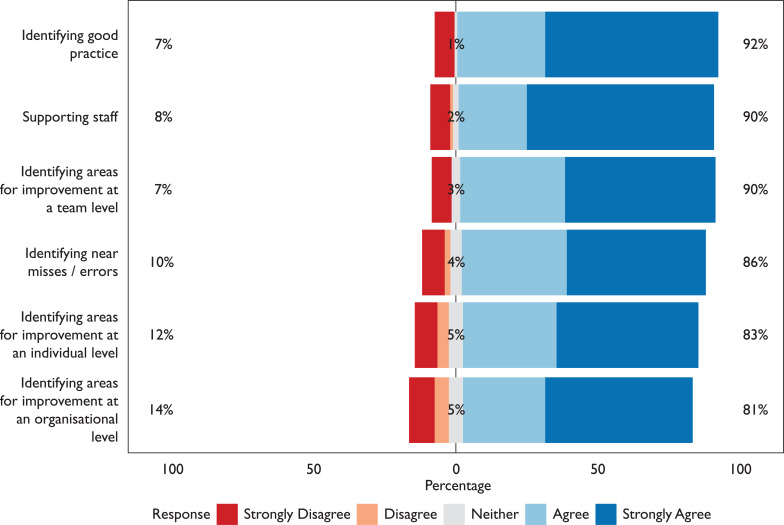
Figure 3. Responses to the statement ‘Debriefing is important in relation to the following areas’.

Note: 101 respondents answered this question, and 30 did not provide a response.

Debriefs were reported as being facilitated by a range of clinical grades, with APs (59%, 60/101) and SPs (33%, 33/101) being the two largest groups, followed by operations managers (21%, 21/101) and paramedics (18%, 18/101). Of the 61 respondents who stated they had facilitated a debrief, 87% (53/61) acknowledged they had not received any formal training, and 79% (49/61) had not utilised a structured framework. Of the 18 who had utilised a structured format, 88% (16/18) believed that having utilised some form of framework the debrief was notably improved, with these individuals utilising a number of approaches, not all validated.

### Post-intervention

A total of 24 debriefs were completed during the trial, performed by eight SPs and two APs. A total of 75 individual clinicians participated in the debriefs. In the post-intervention survey, 85.1% (40/47) of participants responded to questions around the impact and improvement in practice of the debrief they received (Tables 1 and 2).

**Table 1.** Areas of impact identified.

Area of impact	n	% of total respondents
Good practice	37	92.5
Areas for improvement at an individual level	24	60.0
Areas for improvement at a team level	29	72.5
Areas for improvement at an organisational level	15	37.5
Identifying near misses/errors	11	27.5
Supporting staff	33	82.5
I do not feel the debrief identified any of the above areas	1	0.0
Total number of respondents	40	85.1
Did not respond	7	14.9

Nine facilitators provided responses to the post-intervention survey, with 100% reporting that the debrief had identified and shared learning across the areas of decision making, teamwork and communication. In addition, all respondents felt that the debrief identified areas for improvement at an individual level, 88.9% (8/9) at a team level and 55.6% (5/9) at an organisational level.

**Table 2.** Improved practice around areas associated with human factors.

Answer choices	Responses	
	n	%
Situational awareness	23	54.8
Decision making	24	57.1
Teamwork and cooperation	30	71.4
Communication	35	83.3
Total number of respondents	42	89.4
Did not respond	5	10.6

## Discussion

### Learning through debriefs

Emergency medicine is an area of medicine that encompasses the understanding and development of pre-hospital care (Royal College of Emergency Medicine, 2017); however, emergency medicine is ranked the third highest error-prone environment within the NHS (Royal College of Emergency Medicine, 2014). Given this high risk of clinical error, accompanied by the fact that the composition of UK pre-hospital teams is unpredictable in numbers, skillset and experience (Shields & Flin, 2012), opportunities for learning and reducing future error through debriefing should be central to practice (Carthey & Clarke, 2013). Nevertheless, there is no published evidence that appropriately structured and facilitated debriefs are consistently occurring within the UK ambulance services.

Given that the central aims of debriefing are learning for improvement, error reduction and the optimisation of performance (Carthey & Clarke, 2013; NHS Institute for Innovation and Improvement, 2010; Runnacles et al., 2014; Sandhu et al., 2014), it is of particular concern that prior to the intervention 22% (22/100) of respondents did not feel that the identification of learning for improvement was achieved during previous debriefs. Neither was good practice identified as reported by 20% (20/100), suggesting that opportunities to identify, share and embed good practice are being missed.

These figures identify missed opportunities within the debriefing process; however, additionally worrying is that 7–14% (7–14/101) disagreed that debriefs are important for identifying good practice or for learning at individual, team and organisational levels, with an additional 18% (18/101) neither agreeing nor disagreeing with these aspects. This raises the question of what staff perceive as being the core aims of a debrief, and whether this view is due to previous experiences.

### Structured and facilitated debriefs

The Debrief Diamond (Jaye et al., 2015) is structured upon educational theory pertaining to advocacy inquiry, a style of refection which leads to deeper learning through discovering rationales behind mental models that inform decisions and behaviours (Eppich & Cheng, 2015; Kessler et al., 2015).

With human factors forming part of the error chain in 70% of cases in critical incidents and serious untoward events (Royal College of Emergency Medicine, 2014), and the defining critical link between debriefing and human factors being evident (Berwick, 2013; Carthey & Clarke, 2013; Kessler et al., 2015; The National Quality Board, 2013), the inclusion of human factors theory as part of the SPs’ and APs’ education day was crucial. A focus upon four areas associated with team dynamics was delivered: situational awareness, decision making, teamwork and communication (Australian Council for Safety and Quality in Healthcare, 2004; Carthey & Clarke, 2013; Dufresne, 2007).

Of the 24 debriefs completed utilising the Debrief Diamond (Jaye et al., 2015), 93% (37/40) reported the identification of good practice within the debrief, a 13% increase on the pre-intervention position. Regarding areas for improvement at team level, 70% (28/40) reported learning within this area; as a question specific to team level learning was not in the original pre-intervention survey due to a design oversight, this response cannot be compared. However, analysis of improved understanding and practice relating to the four defined areas of human factors (Table 2) evidences positive learning has been achieved in these areas, areas recognised as being sources of error in the fallible human (Carthey & Clarke, 2013).

Of those receiving a debrief in the trial, 89% (34/38) were of the view that the process was improved utilising a structured framework, with 85% (34/40) stating a trained individual facilitating improved the process. Of the nine responding facilitators, none had previously utilised a structured framework, with only one having previously received formal training. Subsequently, 100% (9/9) of facilitators believed the use of a structured framework improved their ability to identify and support learning. Equally important is the acceptability of the tool by the facilitators. Although the responses were limited in number, all agreed that it was an appropriate framework for debriefing.

### Limitations

The low response rate of the pre-intervention survey does raise questions regarding sample bias with the risk of statistical error on analysis (Dillman, Smyth, & Christian, 2014); the results are further compounded by the completion rate and subsequent variation across some questions due to incomplete questionnaires. The surveys were designed so that questions would be skipped by individuals if previous answers identified an inappropriate question due to the respondent’s experience, therefore individual question response rates reflect this variance. Additionally, not all surveys were completed, possibly as a result of crews being called out part way through completion, resulting in further variance across question response rates.

In addition, the Debrief Diamond is a tool validated for use in the simulation environment, and requires further validation to its use in other settings, a fact Jaye et al. (2015) directly acknowledge.

## Conclusion

UK ambulance services appear to be falling behind other healthcare providers in the implementation of systems and practices to identify and address clinical error. This may be addressed, in part, by the identification and implementation of a structured, consistent, validated debriefing framework to support the fostering of a learning culture.

Improvements in identifying good practice and learning opportunities were reported by both clinicians and facilitators in this evaluation, reflecting current evidence that structured and facilitated debriefs support safer care through the identification and subsequent reduction of human error. Consequently, the evaluation of appropriate debrief frameworks to provide consistency and validity to clinical debriefs in the pre-hospital environment should be considered to support safer clinical care.

## Conflict of interest

None declared.

## Ethics

Ethical approval was gained by the trust and university through which this study was being supervised.

## Funding

None.
